# EAACI position paper on occupational rhinitis

**DOI:** 10.1186/1465-9921-10-16

**Published:** 2009-03-03

**Authors:** Gianna Moscato, Olivier Vandenplas, Roy Gerth Van Wijk, Jean-Luc Malo, Luca Perfetti, Santiago Quirce, Jolanta Walusiak, Roberto Castano, Gianni Pala, Denyse Gautrin, Hans De Groot, Ilenia Folletti, Mona Rita Yacoub, Andrea Siracusa

**Affiliations:** 1 Allergy and Immunology Unit, Fondazione 'Salvatore Maugeri', Institute of Care and Research, Scientific Institute of Pavia, Pavia, Italy; 2 Service de Pneumologie, Cliniques de Mont-Godinne, Université Catholique de Louvain, Yvoir, Belgium; 3 Department of Allergology, Erasmus MC, Rotterdam, The Netherlands; 4 Center for Asthma in the Workplace, Hôpital du Sacré-Coeur de Montréal, Centre de Recherche-Pneumologie, Montreal, Quebec, Canada; 5 Allergy Department, Hospital La Paz, Madrid, Spain; 6 Department of Occupational Diseases, Institute of Occupational Medicine, Lodz, Poland; 7 Occupational Medicine, Terni Hospital, University of Perugia, Perugia, Italy

## Abstract

The present document is the result of a consensus reached by a panel of experts from European and non-European countries on Occupational Rhinitis (OR), a disease of emerging relevance which has received little attention in comparison to occupational asthma. The document covers the main items of OR including epidemiology, diagnosis, management, socio-economic impact, preventive strategies and medicolegal issues. An operational definition and classification of OR tailored on that of occupational asthma, as well as a diagnostic algorithm based on steps allowing for different levels of diagnostic evidence are proposed. The needs for future research are pointed out. Key messages are issued for each item.

## Key messages

### Definition and classification

• Occupational rhinitis is an inflammatory disease of the nose, which is characterized by intermittent or persistent symptoms (i.e., nasal congestion, sneezing, rhinorrea, itching), and/or variable nasal airflow limitation and/or hypersecretion due to causes and conditions attributable to a particular work environment and not to stimuli encountered outside the workplace

• Work-related rhinitis may be distinguished into: (1) occupational rhinitis that is due to causes and conditions attributable to a particular work environment (2) work-exacerbated rhinitis that is pre-existing or concurrent rhinitis exacerbated by workplace exposures

### Epidemiology

• Surveys of workforces exposed to sensitizing agents indicate that OR is 2 to 4 times more common than OA, although the contribution of workplace exposures to the general burden of rhinitis remains unknown

• The level of exposure is the most important determinant of IgE-mediated sensitization to occupational agents and OR

• Atopy is a risk factor for the development of IgE-mediated sensitization to HMW agents, but the association with clinical OR due to HMW agents is less well substantiated

### Relationships with occupational asthma

• The majority of patients diagnosed with OA also suffers from OR, which most often precedes the development of OA, especially when HMW agents are involved

• OR is associated with an increased risk of asthma, although the proportion of subjects with OR who will develop OA remains uncertain

### Investigation and diagnostic approach

• Questionnaires and the clinical history have a low specificity for diagnosing OR

• Immunological tests (skin prick tests and specific IgE antibodies) are sensitive but not specific tools for diagnosing OR due to most HMW agents and some LMW agents (i.e., platinum salts, acid anhydrides, and reactive dyes)

• In the presence of work-related rhinitis symptoms, objective assessment using nasal provocation challenges in the laboratory or at the workplace should be strongly recommended

### Management

• Complete avoidance of exposure to the agent causing allergic OR should still be recommended as the safest and most effective therapeutic option

• When complete elimination of causal exposure is expected to induce important adverse socio-economic consequences, reduction of exposure with relevant pharmacotherapy may be considered an alternative approach, especially in workers with a lower risk of developing asthma (e.g., workers without non-specific bronchial hyperresponsiveness, with mild/recent disease or with a short expected duration of exposure); these workers should however benefit from close medical surveillance aimed at an early detection of OA

### Socio-economic impact

• The socio-economic impact of OR is unknown, but is likely to be substantial in terms of work productivity as can be extrapolated from data available for allergic rhinitis in general

### Prevention

• Primary prevention strategies should focus on reducing exposure to potentially sensitizing agents

• Identification and exclusion of susceptible workers is not efficient, particularly when the marker of susceptibility (e.g. atopy) is prevalent in the general population

• Surveillance programmes aimed at an early identification of OR should include periodic administration of questionnaires and immunological tests when available

• Surveillance of workers should focus on the first 2 to 5 years after entering exposure

• The possibility of OA should be carefully evaluated in all workers with OR

### Medico-legal aspects

• Workers with OR should theoretically be considered impaired on a permanent basis for the job that caused the condition as well as for jobs with similar exposures

• Compensation of OR should aim at providing incentives to accommodate workers to unexposed jobs and offering vocational rehabilitation programs in order to minimize the adverse socio-economic consequences of the disease

## Introduction

**Figure 1:**
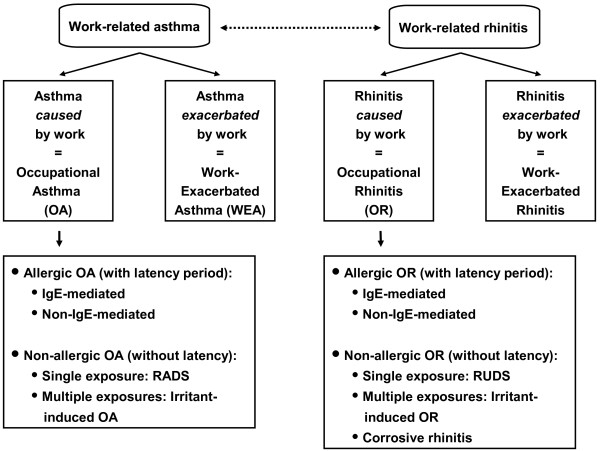
**Parallel classification of occupational rhinitis and asthma**. The Table classifies occupational rhinitis according to the most recent classification of occupational asthma. RADS, Reactive Airways Dysfunction Syndrome; RUDS, Reactive Upper Airways Dysfunction Syndrome).

The health and socio-economic impact of rhinitis, as well as the interaction between upper and lower airways have been emphasized in recent years [1-8]. By contrast, little attention has been paid to occupational rhinitis (OR), although it is increasingly acknowledged that the burden of this condition is largely underestimated in comparison with occupational asthma (OA) [9-12] [see also Figure 1].

There is currently no consensus on the definition and classification of OR. In addition, diagnostic procedures and strategies for the management of subjects with OR remain poorly standardized. This is a particularly important point as an accurate and early recognition of OR in surveillance programs is not only important *per se*, but is also useful in the prevention and early diagnosis of OA.

The purpose of this document was to provide a comprehensive and critical review of available information on the different aspects of OR including diagnostic procedures, management, societal burden, and preventive strategies. The principal objective was to issue key messages and consensus recommendations based on existing scientific evidence and the expertise of a panel of physicians coming from different European and non European countries.

## Definition and classification

The most widely cited definition of rhinitis has been formulated by the International Consensus Report and states that '*rhinitis is defined as inflammation of the lining of the nose, characterized by one or more of the following symptoms: nasal congestion, rhinorrhea, sneezing, and itching*' [1,13]. This International Consensus Report has proposed an operational definition of rhinitis which was based solely on symptoms and would require the presence of '*two or more nasal symptoms for more than one hour on most days*', although these criteria have never been formally validated [14-16].

Most previously published definitions of OR were based **on **the temporal relationship between nasal symptoms and workplace exposure [13,17-20], while a few others also refer to the underlying inflammation [12,21]. However, the major symptoms of OR (i.e., sneezing, rhinorrhea, nasal congestion, and itching) are similar to those of non-occupational rhinitis. Defining OR based only on work-related symptoms would therefore suffer the limitation of being inaccurate [22], as already outlined for OA [23].

In addition, the similarities and tight interactions between rhinitis and asthma [1-8] support the need for homogeneous definitions of OR and OA. The most widely accepted definition of OA refers to the pathophysiological changes that occur in the lower airways, i.e. '*variable airflow limitation and/or bronchial hyperresponsiveness and/or inflammation*' [24,25]. A similar approach cannot easily be translated to OR because: (1) nasal airflow limitation is not always present in OR; and (2) the various methods used for assessing nasal patency, non-specific hyperresponsiveness, and inflammation have not been thoroughly validated [26,27], and (3) these procedures are still largely underused in clinical practice. Nevertheless, considering that inflammatory changes of the mucosa are the common feature of both rhinitis and asthma [1-8,12], the following consensus definition of OR is proposed:

*'Occupational rhinitis is an inflammatory disease of the nose, which is characterized by intermittent or persistent symptoms (i.e., nasal congestion, sneezing, rhinorrea, itching), and/or variable nasal airflow limitation and/or hypersecretion due to causes and conditions attributable to a particular work environment and not to stimuli encountered outside the workplace'*.

The central concept of this broad definition is the *causal *relationship between work exposure and the development of the disease. In addition, this definition is based on demonstrable pathophysiological changes, and it does not place restriction according to the underlying mechanism.

There is accumulating evidence that the workplace environment can induce or trigger a wide spectrum of rhinitis conditions involving immunological and non-immunological mechanisms [16,20,28,29]. These various conditions should be referred to as 'work-related rhinitis' and should be further distinguished according to the clinical features, etiopathogenic mechanisms, and the strength of the evidence supporting the causal relationship.

According to the revised nomenclature for allergy recently recommended by the European Academy of Allergy and Clinical Immunology [30] and the classification of work-related asthma proposed by panels of experts [24,25,31] different types of '*work-related rhinitis*' may be delineated as detailed below and summarized in Figure 1. This review will, however, focus on immunologically-mediated OR, since there is only scarce data on the other forms of work-related rhinitis.

### Occupational rhinitis

#### Allergic OR

Work-related rhinitis symptoms are caused by immunologically-mediated hypersensitivity reactions resulting from antibody- or cell-mediated mechanisms. This entity is characterized clinically by the development of nasal hypersensitivity to a specific occupational agent appearing after a latency period, which is necessary to acquire immunological sensitization to the causal agent. Once initiated, the symptoms recur on re-exposure to the sensitizing agent at concentrations not affecting other similarly exposed workers. The symptoms can be intermittent or persistent according to the frequency and intensity of exposure to the causal agent. In allergic OR, the causal role of occupational agents can be documented on an individual basis through nasal provocation test (NPT), showing reduction of nasal patency, increased volume of nasal secretions, and/or nasal inflammation. Allergic OR encompasses both IgE-mediated OR and non-IgE-mediated OR.

1. *IgE-mediated OR*: Can be caused by a wide variety of high-molecular weight (HMW) agents (i.e. glycoproteins from vegetal and animal origin) and some low-molecular-weight-agents (LMW) for which an IgE-mediated mechanism has been proven, such as platinum salts, reactive dyes, and acid anhydrides.

2. *Non-IgE-mediated OR*: Can be induced by LMW agents (e.g. isocyanates, persulphate salts, woods) acting as haptens for which the allergic mechanism has not yet been fully characterized.

#### Non-allergic OR

This category encompasses different types of rhinitis caused by the work environment through irritant, non-immunological mechanisms. It has been documented that single or multiple exposures to very high concentrations of irritant compounds can lead to transient or persistent symptoms of rhinitis [32-34]. Such cases of acute-onset *'irritant-induced OR' *usually occur without a latency period, although the absence of latency may be obscured when workers are repeatedly exposed to high levels of irritants at work. This entity is quite similar to the situation of '*reactive airways dysfunction syndrome*' (RADS) [25,31,35], so that the term '*reactive upper airways dysfunction syndrome*' (RUDS) has been proposed [36]. Biopsies of the nasal mucosa among individuals with RUDS induced by chlorine have shown non specific pathologic changes (i.e., lymphocytic inflammation of the lamina propria, epithelial desquamation, defective epithelial cell junctions, and increased numbers of nerve fibres) [32]. In these cases of irritant-induced OR, evidence supporting a causal relationship with the workplace can be drawn only from the temporal association between exposure to unusually high levels of irritants and the development of rhinitis symptoms (or other objective indices of the disease).

The term '*irritant*-*induced *OR' may also refer to symptoms of rhinitis reported by subjects repeatedly exposed at work to irritants (vapours, fumes, smokes, dusts) without identifiable exposure to high concentration of irritants. A variety of occupational exposures have been associated with rhinitis symptoms, nasal airflow obstruction, and/or nasal inflammation, usually with a predominant neutrophilic component, including ozone, volatile organic compounds, fuel oil ash, grain and cotton dust, formaldehyde, chlorine, wood dust, thermal degradation products of polyurethanes, and waste handling [12,21]. The symptoms may be experienced only during exposure to irritants at work or may be persistent, presenting both at work and off work.

The term 'corrosive rhinitis' has been used to describe the most severe form of 'irritant-induced OR', which is characterized by permanent inflammation of the nasal mucosa (sometimes associated with ulcerations and perforation of the nasal septum) that may develops after exposure to high concentrations of irritating and soluble chemicals [20,29,37].

### Work-exacerbated rhinitis

Work-exacerbated rhinitis (WER) should be defined as pre-existing or concurrent (allergic or non-allergic) rhinitis that is worsened by workplace exposures [22,29], while the disease has not been caused by the work environment. It is indeed highly likely that rhinitis symptoms can be triggered by a wide variety of conditions at work, including irritant agents (e.g., chemicals, dusts, fumes), physical factors (e.g., temperature changes), emotions, second-hand smoke, and strong smells (e.g., perfumes). Epidemiological surveys have usually found high prevalence rates of work-related nasal symptoms in a variety of workforces, although IgE-mediated sensitization to occupational agents was not detected [22,38-42]) or nasal inflammation was not documented [43-47].

The clinical features of a WER are similar to those of occupational rhinitis, so that the possibility of a WER should be considered only after careful exclusion of a specific sensitization to a workplace agent through appropriate diagnostic procedures. The mechanisms involved in the development of WER have been scarcely explored. The nasal response to irritant stimuli show wide inter-individual variability, and exaggerated reactivity to common chemical and physical stimuli, and is affected by age, gender, and the presence of allergic rhinitis [48-50].

## Epidemiology

### Prevalence and incidence

Although rhinitis is a common condition, the prevalence and incidence of OR in the general population have almost never been specifically investigated. Analysis of incident cases of OR reported to the Finnish Register of Occupational Diseases during the period 1986–1991 showed that occupations at increased risk include furriers, bakers, livestock breeders, food-processing workers, veterinarians, farmers, electronic/electrical products assemblers, and boat builders [51].

Cross-sectional studies have been conducted in various working populations exposed to a wide range of HMW and LMW agents, as recently reviewed [9]. Prevalence rates of OR varied from 2% to 87% in workforces exposed to HMW agents and from 3% to 48% in those exposed to LMW agents (Table 1). Available data indicate that OR is usually 2–4 times more prevalent than OA [9,52]. Prevalence estimates of rhinitis and OR are largely affected by the criteria used for identifying the condition [52]. The incidence of work-related nose symptoms (WRNS) has been investigated in a few prospective cohort studies that are summarized in Table 2[38-41,53,54].

**Table 1:** Prevalence and aetiological agents in occupational rhinitis (adapted from reference [9]).

**Agents**	**Occupation**	**Prevalence (%)**
**High molecular weight agents**		

Laboratory animals	Laboratory workers	6–33

Other animal-derived allergens	Swine confinement workers	8–23

Insects & mites	Laboratory workers, farm workers	2–60

Grain dust	Grain elevators	28–64

Flour	Bakers	18–29

Latex	Hospital workers, textile factory	9–20

Other plant allergens	Tobacco, carpet, hot pepper, tea, coffee, cocoa, dried fruit and saffron workers	5–36

Biological enzymes	Pharmaceutical & detergent industries	3–87

Fish and seafood protein	Trout, prawn, shrimp, crab & clam workers; aquarists & fish-food factory workers	5–24

**Low molecular weight agents**		

Diisocyanates	Painters, urethane mould workers	36–42

Anhydrides	Epoxy resin production, chemical workers, electric condenser workers	10–48

Wood dust	Carpentry & furniture making	10–36

Metals (*platinum*)	Platinum refinery	43

Drugs *(psyllium, spiramycin, piperacillin)*	Health care & pharmaceutical workers	9–41

Chemicals	Reactive dye, synthetic fibre, cotton, persulphate, hairdressing, pulp & paper, shoe manufacturing	3–30

**Table 2:** Incidence of occupational asthma and rhinitis

**Reference/agent**	**Subjects** **N**	**Years/duration** **(yrs) of follow-up**	Incidence of OA* (× 100 person years)	Incidence of OR** (× 100 person years)
Cullinan el al., 1999/laboratory animals [38]	342	1990–1993/2.7	3.5	7.3

Rodier et al., 2003/laboratory animals [39]	387	1993–1995/3.7	2.7	12.1

Draper et al., 2003/laboratory animals [54]	17300	1999–2000/1.0	0.2	0.3

Cullinan et al., 2001/flour [40]		1990–1993/3.3	4.1	11.8

Gautrin et al., 2002/flour [41]		1993–1997/1.4	NA***	13.1

Archambault et al., 2001/latex [53]		1993–1995/2.7	1.8	0.7

*OA: occupational asthma; **OR, occupational rhinitis ; ***NA, not available.

### Risk factors

Exposure, atopy, and smoking have consistently emerged as the main potential determinants for the development of OR.

#### Level of exposure

A dose-response gradient between the level of exposure and IgE-mediated sensitization has been substantiated for various HMW agents, such as laboratory animals [55,56], flour [57,58], insects [59], alpha-amylase [60], and detergent enzymes [61]. However, much of the evidence relates more strongly to immunological sensitization (i.e., the development of specific IgE antibodies) than to clinical OR [9]. Although the relationships between these two outcomes are poorly understood, the development of IgE sensitization to some agents seems to be a strong predictor of rhinitis/asthma symptoms [41,62-64].

#### Atopy

Atopy has been associated with an increased risk of specific sensitization to a variety of HMW agents [9,52]. Atopy is associated with OR due to these agents (Table 1) [41,65,66]. Available studies have provided inconsistent results regarding the relationships between atopy and OR or specific sensitization in workers exposed to LMW agents [9,63] but atopy is unlikely to play a role.

Unlike occupational asthma, the role of genetic factors in the development of OR has never been specifically assessed. [67].

#### Smoking

The relationships between smoking and occupational sensitization, OR, and OA remain controversial [68-70].

#### Non-specific bronchial hyperresponsiveness

There is some evidence from cohort studies [63,71] that non-specific bronchial hyperresponsiveness may be associated with an increased risk for the subsequent development of work-related nasal symptoms.

## Relationships with occupational asthma

Increasing scientific evidence shows that among the general population asthma and rhinitis might be a unique disease with manifestations in different sites of the respiratory system [2,5,7]. The '*united airway disease*' model describes such a relationship as two clinical manifestations of a single disorder. The evidence of this relationship comes from the observation of common epidemiologic [72], physiopathologic, [73], clinical and therapeutic features in both conditions [2,3]. These links have been more frequently observed when considering allergic mechanisms, but the concept of a unique disease has evolved and non-allergic mechanisms may be considered as well.

With regard to the association between rhinitis and asthma of occupational origin, Malo and coworkers have documented that rhinitis symptoms are common among subjects with OA, 92% patients with OA reporting symptoms of OR [74]. The prevalence of rhinitis symptoms was not different for HMW and LMW agents, although the intensity of symptoms was more pronounced for HMW agents. A recent prospective study carried out in patients referred to four tertiary care clinics for possible OA, showed that nasal itching and secretions as well as ocular itching were satisfactory predictors of the presence of OA as confirmed by specific inhalation challenges [75]. However, this likely prediction obtained from questionnaire items applied for HMW but not for LMW agents.

Symptoms of OR have been reported to develop before those of OA in 20 to 78% of affected subjects [22,64,74,76-80]. There is some suggestion that symptoms of OR were more often reported to precede OA in the case of HMW compared to LMW agents [74,77]. A longitudinal study of patients seeking compensation for OR from the Finnish Register of Occupational Diseases, followed via register linkage showed an increased risk of asthma (RR 4.8, 95%CI 4.3 to 5.4) among those with OR compared to subjects with other occupational diseases [81]. The incidence rate ratio for asthma in workers with occupational rhinitis varied across occupations, being highest for farmers and woodworkers [81].

The time-course pertaining to the development of immunological sensitization and nasal or respiratory symptoms related to the onset of exposure has seldom been examined. As for environmental allergens, it can be hypothesized that workers first develop immunological reactivity and subsequent to this, symptoms related to a specific organ (skin, nasoconjunctival, respiratory), this sequence being referred to as the 'allergic march'. Among apprentices in animal health technology [62], development of skin reactivity and nasoconjunctival symptoms mainly occurred in the first two years after starting exposure, confirming the findings of a longitudinal study of animal workers [38], whereas onset of respiratory symptoms was more common in the second and third year of the apprenticeship program. In this study, the predictive value of the development of work-related nasal symptoms on the subsequent development of probable OA was only 11.4% over a 44-months period [62]. An annual surveillance program of workers exposed to laboratory animals (582 workers contributing 2414 person-years) found that the probability of experiencing asthma symptoms by the 11th year of follow-up was 36.7% for workers with animal-related rhinoconjunctivitis symptoms and 5.2% for those without allergy symptoms [82]. By contrast, a 24-month prospective study of apprentice bakers showed that OR could be diagnosed at an earlier stage in only 20% of cases of OA [64].

## Investigation and diagnostic approach

The investigation of OR includes both assessing the presence of rhinitis and demonstrating their work-relatedness. The use of objective methods to assess nasal patency and parameters of inflammation in nasal secretions minimizes patient misclassification.

**Figure 2:**
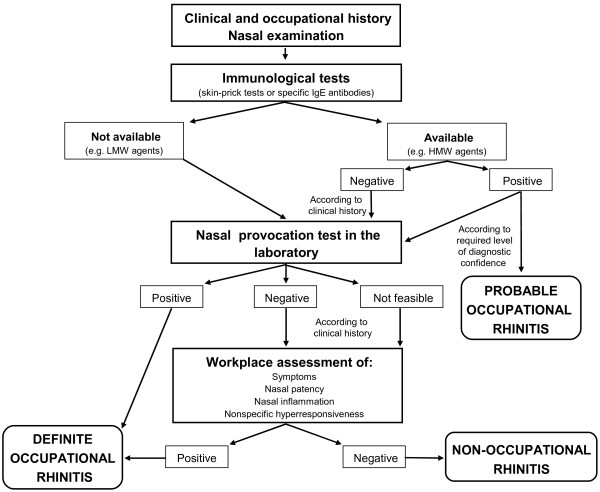
**Diagnostic algorithm**. The figure illustrates the sequential steps for diagnosing occupational rhinitis.

The diagnosis needs to be confirmed by means of objective methods, as misdiagnosis may have substantial social and financial consequences. The different steps involved in the diagnosis of OR are the clinical history, nasal examination, immunological tests (for allergic OR), and nasal provocation tests (NPT) [see Figure 2].

In addition, the possibility of lower airways involvement should be carefully evaluated by means of questionnaire, spirometry, measurement of non-specific airway responsiveness [1,83,84] and evaluation of inflammation by means of exhaled NO [85].

### Clinical and occupational history

Detailed medical and occupational history remains a key step in investigation and diagnosis of OR. The occupational history should aim at collecting a thorough description of current worker's job duties, processes in adjacent work areas, recent changes in work processes or materials, and workplace hygiene conditions. Safety data sheets of the compounds to which the subject is directly and indirectly exposed should be gathered.

One purpose of the medical history when evaluating OR is to establish the timing of nasal symptoms in relation with occupational exposure, as suggested for OA [75,86]. History taking should address the following features: duration of employment at current job before onset of symptoms (latency period); agents, tasks or processes associated with the onset or aggravation of symptoms; improvement away from work (weekends or prolonged holidays).

The clinical history should also gather information on the nature, severity, and impact of rhinitis symptoms. Nasal symptoms reported by workers suffering from OR are similar to those experienced by individuals from the general population with non-occupational rhinitis (i.e. rhinorrhea, sneezing, nasal blockage, and itchy nose). Conjunctival complaints often accompany these symptoms, especially in allergic IgE-mediated OR. [74] A prospective study carried out among a large cohort of animal-health apprentices showed that symptoms such as sneezing, rhinorrhea and itchy eyes (typical of an early temporal reaction after exposure to an allergen) tended to develop early after starting exposure, whereas symptoms such as stuffy nose appeared later [39].

Although an essential step of the diagnostic approach, the clinical history is not specific enough to establish a diagnosis of allergic OR [22,38-41,52].

### Nasal examination

Unlike the lower airways, the nose provides a unique opportunity to visualize macroscopic appearance of the nasal mucosa using anterior rhinoscopy and nasal endoscopy. These techniques, however, do not allow quantitative assessment of nasal changes. Their main value is to rule out other nasal pathologies that may mimic rhinitis or contribute to aggravate nasal obstruction (e.g, septal deviations, nasal polyps) in patients with rhinitis.

### Physiological assessment

#### Nasal patency

Objective methods that can be used for assessing nasal patency during the investigation of OR include rhinomanometry, acoustic rhinometry and peak nasal inspiratory flow (PNIF) [26,87,88]. These techniques share a common great inter-individual variability that limits their applicability in clinical practice. Thus, it is not possible to rely on comparisons with reported values of healthy subjects to make a diagnosis of rhinitis. Nevertheless, the above methods have well-defined reproducibility, whereby their use is justified for evaluating nasal response to NPTs, during which patients act as their own control.

Anterior rhinomanometry measures separately the airway resistance of each nostril. The technique requires relatively little patient instruction and is easy to perform, but measurements are difficult when the nostril becomes occluded. Posterior rhinomanometry assesses the total resistance of nasal airways through an oral catheter in the pharynx, but the technique requires collaboration of the patients [89]. Available studies provide conflicting data regarding the correlation of rhinomanometry with nasal symptoms. The measurement of peak nasal inspiratory flow (PNIF) has been proposed as a simple, inexpensive, and readily available tool for evaluating nasal airway patency [90]. A good correlation between PNIF and rhinomanometry has been shown by some reports [91-93], but PNIF is less sensitive in detecting changes in nasal patency [94]. Serial self-measurements of peak nasal inspiratory flow can be performed to assess airflow obstruction at the workplace. Acoustic rhinometry is a relatively new technique that assesses nasal patency by determining the cross-sectional area and volume of nasal cavities using the reflection of sound waves [89]. This technique is non-invasive, reproducible, and requires little cooperation from the patient [95]. This technique has been useful for demonstrating cross-shift changes in nasal patency in different industries and would be promising for workplace challenges.

#### Nasal inflammation

Inflammatory cells and mediators can be measured in nasal secretions [27]. Nasal secretions can be collected and weighted for quantifying the secretory activity, especially after allergen challenges [96,97]. The use of nasal lavage in clinical practice is still limited due to great interindividual variability and the lack of a standardized and validated method. Accordingly, this technique is more useful in situations where subjects serve as their own controls as it occurs during NPT or exposure at the workplace. Inflammatory cells can also be assessed using nasal biopsies [27], whose applicability is limited by their invasive character, or using nasal scrapings or brush samples, which are simple and relatively painless procedures [98]. In subjects with allergic rhinitis, a good correlation has been found between nasal eosinophils and clinical parameters, including nasal symptoms and nasal patency [99]. More recently, measurement of nasal nitric oxide (NO) has been proposed as a non-invasive marker of nasal inflammation [85,100]. There are currently no data pertaining to the usefulness of measuring nasal NO in the investigation of OR.

#### Non-specific nasal hyperreactivity

Non-specific nasal hyper-reactivity is an important feature of allergic and non-allergic rhinitis and can be defined as an increased nasal response to a normal stimulus resulting in sneezing, nasal congestion, and/or nasal secretion [101,102]. By contrast to bronchial hyperreactivity in asthma, nasal hyperreactivity is not so much documented in occupational rhinitis. Nasal challenge tests with histamine, methacholine [102-104] and cold dry air [105,106] have been proposed as a method to quantify non-specific upper airway hyperreactivity. Histamine and methacholine responsiveness represent different forms of upper airway hyperreactivity [101,102]. Histamine is by far the most commonly used stimulus and hyperreactivity to histamine has been convincingly demonstrated to occur after allergen provocation [107,108]. Methacholine hyperreactivity has been reported to increase after allergen provocation [109] but not all studies reproduced these findings [110]. Intranasal cold dry air has been shown to be superior to histamine challenge in measuring nasal hyperreactivity in nonallergic non infectious perennial rhinitis [105,106].

### Immunological tests

The demonstration of IgE-mediated sensitization to occupational agents can be achieved by means of skin prick test and/or assessment of serum allergen-specific IgE antibodies. However, the sensitivity and specificity of immunological tests have almost never been established in comparison with NPTs. In recent studies, only 42% of subjects with work-related rhinitis and positive skin-prick tests to laboratory animals showed a positive NPT with relevant allergen extracts [52]. Among 47 bakery apprentices who developed work-related rhinitis symptoms over a 2-year period, NPT was positive in the 36 subjects demonstrating IgE sensitization to flour but also in two subjects with negative immunological tests [64]. Positive immunological test may occur in a substantial proportion of exposed asymptomatic individuals [63,111-114], so that the specificity of immunological tests may be lower than their sensitivity. On the other hand, a negative test result makes the diagnosis of OR unlikely, provided that the appropriate allergens have been tested. The major limitation of immunological tests in the investigation of occupational allergy results from the lack of commercially available and standardized extracts for most occupational agents, especially LMW agents.

### Nasal provocation tests

These tests are still considered the gold standard for confirming the diagnosis of OR [88,115-118]. The major rationale for performing NPT is to explore, through a direct observational approach, the causal relationship between exposure to a specific occupational agent and elicitation of the characteristic features of rhinitis. NPTs can be performed either in the laboratory under controlled conditions or at work under natural conditions. The methods that can be used to deliver occupational agents and to measure nasal response during NPTs have been critically reviewed [9,26,27,88,97,115-120] and recommendations have been published by the European Academy of Allergy and Clinical Immunology [119] and the Committee on Objective Assessment of the Nasal Airways of the International Rhinologic Society [88]. The methodology of NPTs is described in detail in Appendix 1. The major limitation of these tests results from the fact that various criteria have been used for defining a positive response [115-118,120-123], but there is a lack of validated comparison between these criteria [96].

### Diagnostic algorithm

Exploring the work-relatedness of rhinitis symptoms remains often difficult, since diagnostic procedures should be adapted to various agents, occupational settings, and available resources. A consensus diagnostic algorithm has been elaborated (Figure 2) by taking into account the following practical constraints: (1) the validity of the tests used for diagnosing OR remains largely uncertain and (2) the level of reliability may vary according to the purpose of the diagnostic evaluation and its expected socio-economic impact.

The first step includes a thorough clinical and medical history, as well as nasal examination. If the suspect of an occupational origin is raised the work-relatedness of the rhinitis should be confirmed by objective methods. The second step includes the evaluation of sensitization to suspected occupational agents through immunological tests (skin-prick tests and/or determination of specific IgE antibodies) for most HMW agents and some LMW agents (i.e. platinum salts, reactive dyes, and acid anhydrides) [124]. A suggestive clinical history plus a positive immunological tests for a well-standardized extract could be considered as probable OR.

The next step involves the objective evaluation of the causal relationship between rhinitis and the work environment through NPTs in the laboratory. If NPT is positive, a definite diagnosis of OR can be established. If NPT is negative, in the presence of a highly suggestive clinical history further evaluations of work-related changes in nasal symptoms, in nasal patency and in nasal inflammation at the workplace or after a period at work are recommended. Assessment at the workplace may be first considered when NPT in the laboratory is not feasible (See Appendix 1).

## Management

The management of OR has a two-sided objective: (1) minimizing nasal symptoms and their impact on the patients' well-being and (2) preventing the development of OA. Therapeutic options include environmental interventions aimed at avoiding exposure to the causal agent and pharmacologic treatment [21]. Due to the tight relationships between OR and OA a closer collaboration between different specialists, ENT, pneumologists and physicians with expertise in occupational medicine and in environmental hygiene may be recommended.

### Environmental interventions

Treatment strategies should focus on avoidance of exposure to the agent causing allergic OR. However, complete avoidance of exposure often implies considerable professional changes for affected workers and is associated with substantial socio-economic consequences [125,126]. Reduction of exposure can be achieved through different ways. Helmet respirators have been shown to be partially effective in reducing the consequences of exposure in patients with OR and asthma due to laboratory animals [127] and to latex [128]. However, the use of these protective respiratory equipments should be considered only for protecting from peak exposures.

### Removal from exposure

Available data indicate that rhinitis could be an early marker of OA. However, having few quantitative estimates [62,81,82] of the long-term risk of asthma among patients with OR makes it difficult to decide whether a worker suffering from this condition should be immediately and completely removed from causal exposure. Therefore, advising a worker with OR to avoid exposure should take into account the following elements.

#### 1. Additional risk factors for the development of asthma

There have been few attempts to determine which patients with allergic rhinitis have the highest risk of developing asthma. A study of patients suffering from allergic rhinitis found that those with non-specific bronchial hyperresponsiveness have a higher risk of asthma [129]. Population studies have confirmed that asymptomatic airway hyperresponsiveness is associated with a more frequent onset of asthma [130-132]. It has been also demonstrated that tboth the severity and duration of allergic and non-allergic rhinitis were important cofactors in determining the risk of developing asthma [133].

#### 2. Possibilities for minimizing adverse socio-economic consequences

Complete removal from offending exposure may be implemented with a lower socio-economic impact when there are possibilities for relocating the worker to unexposed jobs within the same company or when possibilities for job retraining are available [126,134].

#### 3. Possibilities for reducing exposure

In workplaces where the level of exposure can be significantly reduced, maintaining workers at their job may be considered a reasonable alternative [135], provided that workers with OR are submitted to close medical surveillance.

The outcome of OR after environmental interventions or removal from exposure has been seldom specifically investigated. In the few follow-up studies of OA that have evaluated concomitant OR, it has been found that the severity of rhinitis improved substantially after reduction of exposure to platinum salts [136] and latex [135,137]. Nevertheless, there is some suggestion that nasal symptoms do not completely resolve even after complete avoidance [135], although it remains unknown whether allergic OR can lead to persistent functional sequelae. In non occupational setting, allergic rhinitis is not associated with remodelling of the nasal mucosa to the same extent as what has been described in asthmatic airways [138,139]

### Pharmacotherapy and immunotherapy

No studies have addressed pharmacotherapy in OR, either allergic or non-allergic. Nevertheless, analogous to non-occupational allergic rhinitis (AR) it may be suggested that it should be instituted according to evidence-based guidelines, such as those proposed by the Allergic Rhinitis and its Impact on Asthma (ARIA) panel of experts [1]. The effect of non-sedating antihistamines and intranasal corticosteroids medications has almost never been specifically assessed in alleviating work-related symptoms of rhinitis. However, in allergic OR, medications should not be considered a suitable alternative to elimination or reduction of workplace exposure to the sensitising agent.

Several studies have reported some improvement in respiratory symptoms during immunotherapy with purified rodent proteins, wheat flour extracts, and natural rubber latex. However, allergen immunotherapy is currently limited by the unavailability of standardized extracts for most occupational allergens and should be used with caution and close supervision until more data are available [140].

## Socio-economic impact

The socio-economic impact of OR has almost never been specifically evaluated and available data thus relate to other forms of rhinitis. Direct costs attributed to allergic rhinitis seem to be rather modest [141-144]. There is, however, growing evidence that important components of the economic burden of allergic rhinitis are indirect cost resulting from the worsening of associated airway diseases (sinusitis, asthma) [145-147], adverse effects of pharmacological treatment [148-152], disease-related loss of work productivity [151,153,154]. The impact of OR on work productivity has been evaluated in a retrospective cohort study of Swedish bakers [155]. Bakers reported having changed their job because of nasal symptoms more often than control subjects. In a study of Norwegian bakeries, the authors mentioned that, during a 2-year follow-up, 5 of 180 workers had to 'leave their jobs due work-related rhinitis, conjunctivitis and/or skin problems but none as a result of asthma' [22].

There is little information on the psycho-social impact of OR, although it has been increasingly recognized that allergic diseases may impair patients' quality of life [153,156-158]. The negative impact of OR on daily life has been investigated in only one study conducted among greenhouse workers [159].

## Prevention

Primary prevention actions focus on environmental and host risk factors in order to prevent the development of OR. Secondary prevention aims to detect OR at an early stage and to take appropriate actions to minimize its duration and severity. Tertiary prevention is applicable only to patients with established OR (see section on Management). Since OR is acknowledged as a risk factor for the development of OA, the prevention of work-related rhinitis may also provide an excellent opportunity to prevent OA.

### Primary prevention

Epidemiological data indicate that the level of exposure to sensitizing agents is the most important determinant of IgE-mediated sensitization and OR and, by implication, reducing or eliminating workplace exposure to sensitising agents should be the most effective approach for minimizing the incidence of the disease.

#### Controlling exposure at the workplace

Examples of effective prevention resulting from reduction of exposure have been documented in enzyme detergent production [160-162], platinum refining workers [163], laboratory workers [164-166], and health care workers using latex gloves [167-171]. One study found a low rate of sensitization to diphenylmethane diisocyanate in a urethane mold plant that had been designed to minimize MDI exposure [86].

Reducing exposure to safe levels remains, however, quite difficult in field practice, because the threshold level (or dose) of a sensitizing agent that can elicit respiratory reactions varies widely among sensitised workers [172,173]. Little is known regarding the risk of sensitization at low concentrations and the existence of a 'no-effect threshold' [174]. Available information suggest that IgE-mediated sensitization is unlikely to occur below concentrations of 0.5 mg/m^3 ^for flour dust [60,112,174], 0.25 ng/m^3 ^for fungal α-amylase allergens [60], 0.7 μg/m^3 ^for urinary rat allergens [175], and 0.6 ng/m^3 ^for natural rubber latex allergens [176]. However, methods for measuring airborne levels of biological agents are not fully standardized and are not yet easily available for evaluating the efficacy of environmental interventions [177].

#### Identification of susceptible workers

The positive predictive values of available susceptibility markers are too low for screening out potentially susceptible individuals [178,179]. This is particularly true in the case of atopy, which is a highly prevalent trait in the general population. Excluding atopic individuals from jobs entailing exposure to HMW allergens would reduce dramatically the number of potential new employees and would be unduly discriminatory. In addition, there is a role for better education on the risk of sensitization of those attending vocational schools [178].

### Secondary prevention

The short latency period for the incidence of OR indicates the need for surveillance of individuals at risk in the very first years of exposure [180]. Surveillance programs should be implemented during vocational training, since sensitization to occupational allergens and work-related nasal symptoms can develop at that time [39,41,53,71,181-184].

Medical surveillance programmes should include the following components [178,185,186]: (1) pre-placement and periodic administration of a questionnaire aimed at detecting work-related symptoms; (2) detection of sensitization to occupational agents by means of skin prick tests or serum specific IgE antibodies when these tests are available and standardized; (3) early referral of symptomatic and/or sensitized workers for specialized medical assessment, including NPT in the laboratory and/or at the workplace [52]; and (4) investigation of possible asthma in all workers with confirmed OR. Unfortunately, the effectiveness of secondary prevention has been studied in few settings and it is often difficult to distinguish the beneficial effects attributable to medical surveillance from those arising as a result of concurrent interventions [162]. In addition, the predictive value of immunological tests for the development of OR and OA may be high for some agents (e.g. platinum salts or acid anhydrides) [187,188], but much lower for other occupations [62].

## Medico-legal aspects

### Assessment of impairment/disability

Considering that persistence of exposure to agent causing allergic OR, will lead to worsening of the disease and is associated with a risk of asthma, patients with ascertained OR should, theoretically, be considered impaired on a permanent basis for the job that caused the condition as well as for jobs with similar exposures. Evaluating the level of functional impairment due to OR is hampered by the absence of reference values for physiological tests. Impairment may take into account the fact that OR may be associated with the development of nasal responsiveness to a variety of physical and chemical stimuli [48,49,101,189] and co-morbid conditions, including olfactory dysfunction, sinusitis, and sleep disorders [190-192]. Assessment of disability should be based on the severity of symptoms and their impact on global health status and quality of life. Quality of life can be assessed using validated instruments [156-158,193-196], although these questionnaires are not currently applicable for use as a clinical tool in individual patients. A classification of rhinitis severity into "mild" and "moderate/severe" has been proposed by the Allergic Rhinitis and its Impact on Asthma (ARIA) based on the impact of symptoms on sleep and daily life [1]. Rhinitis should be considered "moderate/severe" when symptoms are troublesome or when they are associated with sleep disturbance or impairment in daily activities, including work, school, leisure and sport. Visual analogue scales (VAS) have also been recommended by the European Consensus on Rhinosinusitis and Nasal Polyps [197] and the American Taskforce on Practice Parameters [198] for quantifying rhinitis and rhinosinusistis symptoms. According to the latest European Position Paper on Rhinosinusitis and Nasal Polyps 2007 the severity of rhinitis can be divided into mild, moderate and severe based on total severity visual analogue scale (VAS) score (0–10 cm): mild (VAS 0–3), moderate (VAS >3–7), severe (VAS >7–10) [199]. The simple ARIA grading system of rhinitis severity have been shown to correlate with impairment in quality of life, quality of sleep, and work productivity [192,200], VAS score [201], and health care utilization [202].

### Compensation

Policies governing compensation of OR vary widely from one country to another (Additional file 1). These differences are due to a number of factors, including administrative regulations and different ways of defining OR, determining causality, and evaluating the level of disability. The criteria used for determining eligibility for compensation are not uniform. For instance, compensation may be restricted to a list of agents recognized as causing OR. The major issue is that these lists are not updated in a timely manner according to scientific evidence. Relying on clinical history or on immunological tests for establishing causality may lead to over- or under-compensation of OR. In some countries, such as Finland, the causal relationship between rhinitis and the workplace should be objectively documented using NPT [97]. Disability resulting from OR is usually rated from 5 to 10% in European countries (Additional file 1), but it is rated higher in some countries such as South Africa (up to 20% if there is a need for medication to control symptoms). Depending on countries' regulations, compensation may cover different aspects: physiological impairment, work disability, loss of income, health care costs, and professional retraining. Available data for OA indicate that the economic consequences of allergic occupational diseases are determined mainly by socio-demographic factors, while the severity of the condition has only a minimal effect [126]. Accordingly, it is not surprising that compensation based on physiological impairment does not adequately offset the financial consequences of the disease [126]. There is now a growing consensus that compensation systems should be directed at accommodating workers to unexposed jobs within the same company and to offer structured rehabilitation programmes when required [179]. Affected workers should also benefit from adequate wage replacement during the period of professional retraining.

### Unmet needs and research areas

#### Definition and classification

• Characterization of the clinical features and pathophysiological mechanisms of 'non-allergic OR' and 'work-exacerbated rhinitis'

#### Epidemiology

• Quantification of the contribution of OR to the general burden of rhinitis in the general population

• Development and validation of an international questionnaire to identify OR in epidemiological surveys and clinical practice

• Further characterization of the role of atopy and smoking in the development of OR

• Elucidation of the interaction between upper and lower airway responses to sensitizing and irritant agents in the workplace

#### Diagnosis

• Standardization of occupational allergen extracts for skin prick tests and for assessment of serum specific IgE

• Standardization and validation of techniques used for measuring non-specific nasal hyperreactivity, nasal airflow, and nasal inflammation

• Development and standardization of nasal provocation tests, including standardization of end points, evaluation of the role of nasal NO measurement, and identification of the most useful biological markers of nasal response

#### Management

• Specific assessment of the impact of OR in terms of QoL and economic burden in order to evaluate the cost-effectiveness of therapeutic and preventive interventions

• Prospective assessment of the efficacy and safety of immunotherapy with occupational agents in controlling rhinitis symptoms and preventing the development of OA

#### Prevention

• Identification of parameters influencing the prognosis of OR

• Assessment of the effects of environmental interventions on the clinical and physiological indices of rhinitis, such as the level of non-specific nasal hyperresponsiveness, and nasal inflammation

• Assessment of the impact of environmental interventions on the development of OA in subjects with OR

#### Compensation

• Definition of consensus criteria for grading impairment/disability resulting from OR

## Abbreviations

AR: Allergic Rhinitis; ARIA: Allergic Rhinitis and its Impact on Asthma; HMW: High molecular weight; LMW: Low molecular weight; NA: Not available; NO: Nitric oxide; NPT: Nasal provocation test; OA: Occupational asthma; OR: Occupational rhinitis; PNIF: Peak nasal inspiratory flow; RADS: Reactive airways dysfunction syndrome; RUDS: Reactive upper airways dysfunction syndrome; VAS: Visual analogue scale; WER: Work-exacerbated rhinitis; WRNS: Work-related nose symptoms.

## Competing interests

The authors declare that they have no competing interests.

## Authors' contributions

This Position Paper is the result of the collaboration of a panel of experts who contributed to the document according to their different experiences and competences. GM designed the paper, coordinated the various contributions, revised and edited the manuscript, and wrote the Chapters Introduction, Definition and Classification, Unmet Need and Research Areas; OV wrote the Chapters Management and Socio economic impact and contributed to the final revision of the manuscript; RGvW and HdG wrote the Chapter Investigation and diagnostic approach; JLM, RC and DG wrote the Chapter Relationship with Occupational Asthma; LP and GP wrote the Appendix Methodology of Nasal Provocation Tests; SQ wrote the Chapter Prevention; JW wrote the Chapter Medico-legal aspects; AS and IF wrote the Chapter Epidemiology; MRY participated in the revision and editing of the manuscript. All authors participated in the definition of the Diagnostic Algorithm and Key Messages. All authors read and approved the final manuscript.

## Appendix 1

### Methodology of nasal provocation tests

#### Precautions

NPT should always be carried out in specially equipped facilities, by trained personnel, and under close medical supervision. In most subjects, the tests can be performed on an outpatient basis, restricting hospitalization to subjects who have severe late reactions [119]. Contra-indications to NPT include pregnancy, recent infectious rhinitis or sinonasal surgery, atrophic rhinitis, and severe asthma [116,117]. Medications known to interfere with nasal response should be withdrawn according to their duration of action [116,117]. The best time to perform a NPT is in the morning in order to limit the effects of daily-life stimuli (fumes, cold air, spicy foods, and exercise). Baseline assessment of symptoms and nasal functions should be performed after adaptation to room temperature for 30 minutes.

It is essential to ensure that the nasal response is specific to the tested occupational agent by performing a control test [97]. These control or sham tests make it possible to detect irritant or non specific hyperresponsiveness. The control substance is selected according to the nature of the occupational agent suspected of causing OR, for instance, diluent for NPTs with aqueous allergen solutions, lactose powder for NPTs with agent in powder form (flour, drugs, persulphates, etc.), pine dust for NPTs with wood dusts. New causal agents have to be tested in control subjects in order to ascertain the specificity of the nasal response.

### Methods of exposure to occupational agents

#### Exposure in the laboratory

Exposure may take place differently according to the nature of the agents;

a) Water soluble HMW agents can be administered as aqueous solutions of allergens. Purified and standardized allergenic extracts should be used for NPTs when such reagents are available. Alternatively, extracts may be freshly prepared into saline (phenolated) solutions [97,203]. In these settings, the level of skin reactivity to the extract can be used as a guide for determining the initial concentration that will be delivered to the patient, which will be 10 times more diluted than the concentration eliciting a skin reaction. In case of negative skin test, the initial dose of allergen should be in the range of 1:10.000 to 1:5000 wt/vol or 50 to 100 PNU [116,117,119]. Aqueous solutions can be delivered using metered dose devices (sprays) as recently reviewed [118] or by means of nebulizers generating aerosols according to dosimeter or tidal breathing protocols. Challenges may regard only one or both nostrils. The latter could avoid the influence of the nasal cycle when monitoring nasal patency [88]. Using allergen solutions offers the advantage of delivering quantified doses and being a reproducible technique. The limitation of this method is that the delivered extract may not represent the native allergens due to purification and extraction procedures, which may potentially lead to falsely negative test. Other methods of exposure such as syringes, pipettes, paper discs, and cotton pads [117] should be discouraged because they do not reproduce natural exposure [118].

b) For all other agents, exposure can be produced in various ways, depending on the chemical properties and the physical state of the agent suspected of causing OR. The agent may be delivered as an aerosol, vapour, gas or dry particles by reproducing as much as possible the conditions of exposure occurring at the workplace. In some cases exposure may be obtained by asking subjects to reproduce their usual work under close supervision [204]. All these tests should be performed in specifically dedicated challenge rooms. Ideally, the concentration of the agent should be controlled and maintained below permissible threshold levels. The level of total and respirable dust and, in some cases (e.g. isocyanates), the concentration of chemicals can be continuously monitored and modified during NPTs. The duration of challenge exposure should be gradually increased under close monitoring of nasal response. Subjects should be exposed for up to 2 hours before the test can be considered negative [204,205].

#### Workplace exposure

Workplace challenges may be considered when exposure to specific suspected agents is not possible, which may occur in the following settings: (1) no sensitizing agent has been firmly identified at work, (2) multiple potentially sensitizing agents are present at the workplace, or (3) the conditions of exposure at work cannot be reproduced in the laboratory (e.g. complex industrial processes) [206] 4) NPTs in the lab are not feasible for unavailability of equipped facilities. During these tests, the worker performs his or her usual tasks, and indices of nasal response are recorded before, during and after one (or several) work shift(s) with a time-schedule similar to that used during laboratory NPTs. Each patient should be compared to itself during a control day in order to ascertain sufficient reproducibility of outcome parameters. NPT at the workplace can also be a useful tool to investigate irritant-induced OR and work-exacerbated rhinitis [207,208].

### Assessment of nasal response

NPTs may induce immediate and/or late response. The European Academy of Allergy and Clinical Immunology's recommendation for monitoring of nasal response entails assessment at 5, 10, 20, 30, 45, 60 minutes post-exposure, and then every hour for 10 hours after the end of the challenge exposure [119]. Several parameters (see clinical assessment) can be used for assessing nasal responses during NPT, including [9,26,27,88,115-122,209] symptoms [(i.e. compound symptom scores [120-122], visual analogue scales (VAS)] [199] nasal patency (i.e., using rhinomanometry, acoustic rhinometry and/or peak nasal inspiratory flow), and nasal inflammatory response (i.e., volume of nasal secretions, eosinophil counts and concentrations of mediators in nasal secretions). Each of these methods has its own advantages and limitations, and their reproducibility has not been studied in large scale studies. There is general agreement that both subjective and objective indices must be considered, but unlike bronchial provocation tests, end-points for NPT are not standardized nor validated. Most frequently, the assessment of the response is made by measuring symptom score and changes in nasal patency [115,120]. There is, however, accumulating evidence that assessment of inflammatory cells (especially, eosinophils) and mediators of inflammation (e.g. eosinophil cationic protein, tryptase) in nasal secretions could increase the specificity of NPTs with HMW [123,210-212] and LMW [213,214] agents and could be helpful for minimizing patient misclassification. A recent study found that NPT with common allergens induced a decrease in the level of nasal NO, which was followed by an increase at 7 and 24 hours post-challenge [215]. The role of nasal NO as a biomarker of airway inflammation during NPTs with occupational agents requires further investigation.

### Pitfalls

A false-negative response on NPTs may occur if the wrong agent has been used, if the exposure conditions are not comparable with those encountered at the workplace, if the patient has been away from work for a long time [216] or if the patient is under nasal steroid treatment.

The most frequent reason for false-positive results in the measurements of nasal patency during NPTs is the effects of the nasal cycle. Other potential causes of false-positive results include a general hyperreactivity of nasal mucosa resulting from preceding exposure to allergens or irritants and episodes of rhinosinusitis ('*nasal priming*') [118,217-219].

## Supplementary Material

Additional file 1 **Table 3**. Compensation for occupational rhinitis in different countries. Click here for file
