# Supplementary material for: EAACI position paper on occupational rhinitis
Source: Respir Res. 2009 Mar 3;10(1):16. doi: 10.1186/1465-9921-10-16 (PMC2654869; doi:10.1186/1465-9921-10-16)
Supplement: Additional file 1 — Table 3. Compensation for occupational rhinitis in different countries. [file 1465-9921-10-16-S1.doc]

**Table 3.** Compensation for occupational rhinitis in different countries.

|  | **Belgium** | **Denmark** | **France** | **Ireland** | **Italy** | **Nederland** | **Portugal** | **Spain** | **Finland** | **Poland** | **United Kingdom** | **Deutschland** | **Austria** | **Luxembourg** |
| --- | --- | --- | --- | --- | --- | --- | --- | --- | --- | --- | --- | --- | --- | --- |
| **List of agents** |  | + | + |  |  |  |  | + |  |  | + | + |  |  |
| **List of occupational diseases** |  |  |  | +^#^ |  |  |  | + |  | + |  |  |  |  |
| **Open system** | + | + |  |  | + | + | + |  | + | + |  |  |  |  |
| **Determinants of the degree of disability** | 1 |  | 2 |  |  | 2 |  |  | + |  | + |  |  |  |
| **Needs of medication** |  | + |  | + | + |  | + |  | + |  | + |  |  | + |
| **Symptoms at work** | + | + | + | + | + |  | + |  | + |  | + |  |  | + |
| **Worker’s possibilities to avoid further exposure** |  | + |  |  |  |  |  |  |  |  |  |  |  |  |
| **Degree of disability** | NA* | 5-10% | <10% | NA | <3% | NA | 10-20% | NA | 10% | <15% | NA | NA | NA | NA |

^#^  rhinitis is not mentioned, but can be recognised under ‘Inflammation or ulceration of the mucous membrane of the respiratory passages or mouth produced by dust or liquid or vapour’

^1^ also nasal dyspermeability, nasal septal perforation, problems of smell, nasal bleeding and various other symptoms

^2^ all these parameters may be taken into account depending on the individual case; none are obligatory

NA, not available
